# Nubia’s mother: being pregnant in the time of experimental vaccines and therapeutics for Ebola

**DOI:** 10.1186/s12978-017-0429-8

**Published:** 2017-12-14

**Authors:** Séverine Caluwaerts

**Affiliations:** 1 Medical Department, Médecins Sans Frontières Operational Centre Brussels, Brussels, Belgium; 2 Institute of Tropical Medicine, Antwerp, Belgium

**Keywords:** Pregnancy, Ebola, West Africa epidemic, Ring vaccination, Favipiravir, ZMapp

## Abstract

During the 2014–2016 Ebola epidemic, Médecins Sans Frontières (MSF) treated Ebola-positive pregnant women in its Ebola Treatment Centers (ETCs). For pregnant women with confirmed Ebola virus disease, inclusion in clinical vaccine/drug/therapeutic trials was complicated. Despite their extremely high Ebola-related mortality in previous epidemics (89–93%) and a neonatal mortality of 100%, theoretical concerns about safety of vaccines and therapeutics in pregnancy were invoked, limiting pregnant women’s access to an experimental live attenuated vaccine and brincidofovir, an experimental antiviral. Favipiravir, another experimental antiviral, was made available to pregnant women only after extensive negotiations and under a ‘Monitored Emergency Use of Unregistered and Experimental Interventions’ (MEURI) protocol. This paper describes the case of a pregnant woman who presented to the ETCs near the end of the Ebola epidemic in Guinea. The pregnant patient was admitted with confirmed Ebola disease. She was previously denied access to potentially protective vaccination due to pregnancy, and access to experimental ZMapp was only possible through a randomized clinical trial (presenting a 50% chance of not receiving ZMapp). She received favipiravir, but died of Ebola-related complications. The infant, born in the ETC, tested positive for Ebola at birth. The infant received ZMapp (under MEURI access outside of the clinical trial), an experimental drug GS5734, and a buffy coat of an Ebola survivor, and survived. Though the infant did have access to experimental therapeutics within 24 h of birth, access to other experimental compounds for her mother was denied, raising serious ethical concerns.

## Case background

At the end of the 2014–2016 West-Africa Ebola epidemic [1], a 25-year old woman, reportedly seven months pregnant, tested positive for Ebola virus disease in Forécariah province, Guinea. She was a follow-up household contact of a known Ebola patient who had died of the disease. At that moment, protective vaccination of Ebola-positive patient contacts with a potentially highly effective live vaccine was available [2]; however, because the woman was pregnant she had not been eligible for vaccination. Pregnancy was an exclusion criterion for vaccination during and after the vaccination trial, despite 90% mortality of pregnant women in previous Ebola Zaire strain epidemics according to available data [3, 4]. The patient also had a very high Ebola viral load, which further increased her mortality risk. The pregnant woman was admitted to an Ebola Treatment Center (ETC) managed by Médecins Sans Frontières (MSF).

At the time of the patient’s admittance to the ETC, a randomized clinical trial of the experimental ZMapp (Mapp Biopharmaceuticals) was ongoing in Guinea and in several other countries [5]. Pregnant women were eligible for inclusion in this trial in which patients were randomly allocated to either receiving only standard supportive care or to receiving the experimental ZMapp in addition to standard supportive care. MSF was not involved in the ZMapp trial. In all Ebola therapeutic trials where MSF was involved, patients received the potentially active drug and comparison was done with historical controls. This is linked to the organization’s belief that every patient infected by a disease with a mortality as high as Ebola should have access to potentially active therapeutics. MSF tried to obtain ZMapp for the pregnant patient outside of the randomized clinical trial because MSF thought that it was unethical to permit a 50% chance of denying this patient from receiving potentially life-saving treatment considering her extremely high chance of dying. Additionally, in the case of this patient, randomization for the purposes of the trial was irrelevant: finding similar patients with corresponding characteristics (pregnancy history, viral load, et cetera) given the epidemiologic situation at that time was very unlikely, so she would have been a complete outlier in the trial. Moreover, she was among the last cases of the epidemic.

ZMapp outside clinical trial was refused. The decision was then made to administer favipiravir, an experimental antiviral that had shown limited success in previous small human studies. In agreement with the company (Toyama Chemical of Japan), use of favipiravir in pregnant Ebola-positive patients was permitted under ‘Monitored Emergency Use of Unregistered and Experimental Interventions’ (MEURI), a concept developed by a WHO convened ethics panel in October 2014) [6]. Four days after admission, the patient went into spontaneous labor and delivered a baby girl of 2800 g, Nubia (permission of the father to use the infant’s name). The patient deteriorated after delivery and died seven hours later of postpartum hemorrhage (PPH) and disseminated intravascular coagulation as a consequence of Ebola, despite receiving oxytocin and misoprostol as treatment for PPH. Nubia also tested positive for Ebola. For the infant, MSF obtained ZMapp outside of the clinical trial without difficulty; Nubia received the first dose the day after her birth. In all, she received four doses of ZMapp, GS5734 (an experimental broad-spectrum antiviral), and white blood cells (buffy coat) of an Ebola survivor; all medications were accessed under MEURI. Nubia recovered and survived [7].

## Ethical discussion

1. Pregnant women were excluded from ring vaccination against Ebola.
  Nubia’s mother contracted Ebola in October 2015. At that time, it was clear that the rVSV ZEBOV live attenuated vaccine was potentially very highly protective against Ebola (the first results were published in August 2015 [2]). While there was a risk of potentially causing harm if the patient were vaccinated—no published data existed on the effects of the vaccine in pregnancy—the vaccine could have potentially prevented her from becoming infected with Ebola. Notably, in the original vaccination trial (Ebola ça Suffit, [2]) pregnancy testing for women of reproductive age was not mandatory before inclusion in the trial and some women in early pregnancy were accidentally vaccinated but analysis of those pregnancies is still ongoing [8].
2. Nubia received access to experimental interventions outside of clinical trials, while her mother did not.
  Nubia’s mother could not gain certain access to ZMapp despite her very poor prognosis (MSF wanted her to receive the drug, but the center had refused access to ZMapp outside of the clinical trial; enrollment in the trial would have meant a 50% chance of receiving only supportive care). Nubia herself received ZMapp a few hours after her birth through MEURI; the infant was not required to be enrolled in the clinical trial in order to receive the drug. Nubia’s mother was denied a potentially beneficial drug while Nubia received the drug without delay. Furthermore, the infant also received the experimental drug GS5734 [7]. Nubia was only the second human in the world to receive this experimental medication, while her mother—who was part of a known Ebola transmission chain and who developed symptoms 10 days after exposure to Ebola a few weeks earlier—was not offered access to an experimental vaccine. It seems that the infant’s health needs were “privileged” compared to the health needs of her mother.
3. Access to experimental compounds for pregnant women was complicated, even for a disease like Ebola with a mortality of more than 50%.
  For favipiravir, only after extensive negotiations between MSF and the manufacturer was MEURI access allowed. Even though the manufacturer was open to the idea of pregnant women being included in the original favipiravir JIKI-trial, the manufacturer’s insurer did not want to provide insurance for pregnant women. In the trial of brincidofovir, another experimental antiviral, in Liberia (stopped prematurely after four patients were included and this due to the epidemiological situation—no new Ebola cases at that time), the manufacturer did not allow the use of the drug in pregnant women [9].

## Conclusions

Access to vaccines and experimental drugs for pregnant women in the 2014–2016 West Africa Ebola epidemic was complicated; for some products, access was simply not allowed by the pharmaceutical companies who produced the drugs/vaccines. Pregnant women did not have access to potentially protective live attenuated vaccines; access to favipiravir was allowed only after extensive negotiations. Access to ZMapp for Nubia’s mother was possible only in a clinical trial setting (with a 50% chance of receiving the only supportive care) while Nubia herself received ZMapp without delay immediately after birth, outside of the clinical trial.

These challenges have yet to be solved. In the event that another Ebola outbreak occurs in the near future, pregnant women still do not have access to protective vaccines, and access to therapeutics remains especially complex. It seems ethically unjust that being pregnant could limit access to potentially life-saving treatment or prevention for a disease with more than 50% mortality, like Ebola.

## Abbreviations

ETC: Ebola Treatment Center
MEURI: Monitored Emergency Use of Unregistered and Experimental Interventions.
MSF: Médecins Sans Frontières.
PPH: postpartum hemorrhage.
